# Acute bilateral ureteric obstruction after Deflux injection: a case report

**DOI:** 10.1093/jscr/rjaf1071

**Published:** 2026-01-15

**Authors:** Yaser M Ata, Muthana Al-Salihi, Kholoud Al-Abassi, Tariq Abbas, Joao L Pippi Salle

**Affiliations:** Department of Surgery, Urology Section, Hamad Medical Corporation, Doha, Qatar; Sidra Medicine, Doha, Qatar; Hamad Medical Corporation, Doha Qatar; Weill Cornell Medicine - Qatar, Doha, Qatar; Department of Surgery, Urology Section, Hamad Medical Corporation, Doha, Qatar; Weill Cornell Medicine - Qatar, Doha, Qatar; Pediatric Urology Section, Sidra Medicine, Doha, Qatar; College of Medicine, Qatar University, Doha, Qatar; Division of Urology, The Hospital for Sick Children (SickKids), Department of Surgery, University of Toronto, Toronto, ON, Canada

**Keywords:** vesicoureteral reflux, Deflux injection, ureteral obstruction, pediatric urology, recurrent urinary tract infection, hydronephrosis, double-J stent, renal scarring

## Abstract

Vesicoureteral reflux (VUR) is a prevalent cause of recurrent urinary tract infections and renal scarring in pediatric patients, managed with conservative measures or surgical intervention. Endoscopic sub-ureteral bulking agent injection provides a minimally invasive option. We present a 7-year-old boy with severe bilateral VUR who developed acute bilateral ureteral obstruction following dextranomer/hyaluronic acid (Deflux) injection. Despite an unremarkable early postoperative course, he developed severe abdominal pain, vomiting, anuria, elevated creatinine, and worsening hydronephrosis. Emergency cystoscopy with bilateral double-J stent placement relieved the obstruction. Significant inflammatory masses at the uretero-vesical junction markedly improved following stent removal. The patient remained asymptomatic without hydronephrosis after 5 years. This case highlights the uncommon but significant risk of ureteral obstruction following endoscopic bulking agent injection and emphasizes the need for careful post-procedural monitoring.

## Introduction

Vesicoureteral reflux (VUR) is a common urological disorder in pediatric patients that may result in recurrent urinary tract infections (UTIs) and renal scarring [1, 2]. Endoscopic intervention with dextranomer/hyaluronic acid (Deflux) has been a prevalent minimally invasive alternative for the management of VUR [3, 4]. Deflux subureteric injection effectively prevents UTIs and kidney damage; nonetheless, problems including ureteral obstruction have been documented, [4, 5] necessitating interventions such as ureteric stenting [6]. Prior research underscores the occurrence of delayed-onset ureteral blockage after to Deflux injections, emphasizing the necessity for vigilant monitoring [7]. This report details a case of a juvenile patient who experienced acute bilateral ureteral obstruction following Deflux subureteric injections, highlighting the necessity of identifying and addressing this complication in children receiving endoscopic treatment for VUR.

## Case presentation

**Figure 1:**
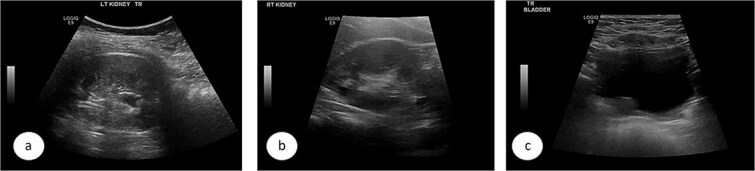
Ultrasound images showing the left (a), and right (b) kidneys and the urinary bladder (c). VCUG is showed bilateral grade 3 VUR (d). DMSA images are shown in (e).

**Figure 2:**
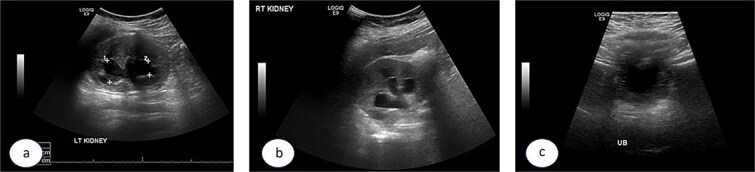
Post Deflux injection ultrasound images showing the left (a), and right (b) kidneys and the urinary bladder (c).

A 7-year-old child was referred to the pediatric urology clinic due to a history of recurrent febrile urinary tract infections and urge incontinence, accompanied by constipation (Bristol 2). These symptoms persisted despite meticulous conservative management, including urotherapy. Ultrasound (US) revealed bilateral pelviectasis and voiding cystourethrogram (VCUG) revealed bilateral Grade 3 VUR and a mildly trabeculated bladder wall, whereas the urethra appeared normal (Fig. 1a–d). A DMSA renal scan verified bilateral renal scarring, revealing differential renal function of 40% in the right kidney and 60% in the left kidney (Fig. 1e). Endoscopic intervention was warranted, and 1 ml of Deflux was injected on the left side and 0.6 ml on the right side, successfully creating an adequate mound. The patient was discharged in stable condition for several hours following surgery, despite being anuric. Subsequently, the patient experienced intense abdominal pain and was admitted to the emergency department. The physical examination was uneventful, except for moderate hypertension. The urinalysis revealed microscopic hematuria. His urea level was 16 mmol/L, and creatinine rose to 126 umol/L from a baseline of 40 umol/L. Post operative US at emergency room demonstrated increased bilateral hydronephrosis (Fig. 2).

**Figure 3:**
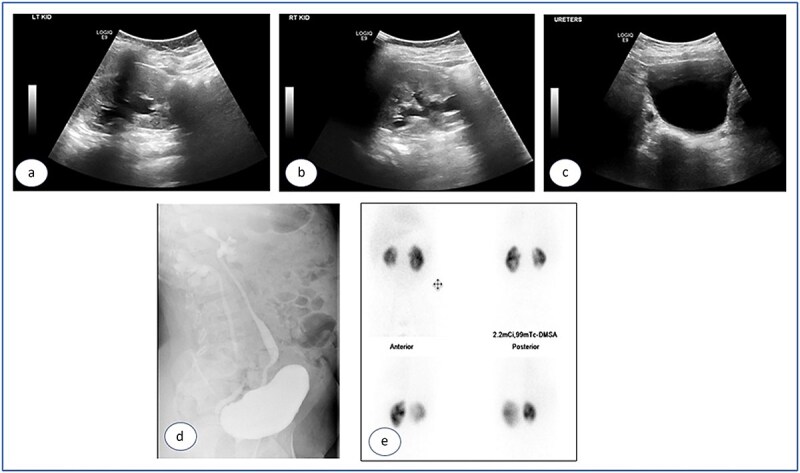
Ultrasound images taken 1 month after the removal of both double J stents, showing no hydronephrosis in either kidney—the left (a) and right (b)—and urinary bladder with Deflux in place (c).

Urgent cystoscopy was performed, and bilateral double-J stents were placed. Both orifices were observed, accompanied by substantial inflammatory masses surrounding them, disproportionate to the initially injected mounds; yet, they did not obstruct the smooth passage of the stents. The patient recovered satisfactorily and was discharged with normal diuresis. One month later, the right double-J stent was extracted, demonstrating a substantial reduction of the inflammatory masses at the uretero-vesical junction. Two months post injection the left stent was removed and, at that point, no inflammatory masses were present. The mucosa appeared normal, exhibiting significant bulges at the Deflux injection sites. Subsequent ultrasound examinations revealed the absence of hydronephrosis bilaterally (Fig. 3a–c), accompanied by normal levels of creatinine and urea. The prophylactic antibiotics were discontinued and the patient was monitored for 5 years, remaining asymptomatic with no more urinary tract infections.

## Discussion

Our case illustrates an uncommon complication post injection of Deflux in a patient with VUR secondary to refractory voiding dysfunction. VUR is a disorder characterized by the retrograde flow of urine from the bladder into the ureters or renal collecting system. It may be categorized as primary, stemming from congenital anomalies at the ureterovesical junction (UVJ), or secondary, arising from elevated intravesical pressure that surpasses the UVJ’s valve mechanism, resulting in reflux [1, 2]. Primary VUR frequently presents without symptoms, complicating the accurate assessment of its prevalence. It is believed that roughly 1% of all children, 10%–20% of infants with prenatal hydronephrosis, and 30%–50% of children presenting with urinary tract infections have VUR [3, 4]. VCUG is the definitive method for diagnosing VUR, categorized into five categories of reflux. Grade 1 is characterized by contrast reaching solely the ureter, whereas grade 5 is distinguished by significant dilatation of the renal pelvis and calyces, accompanied with ureteral tortuosity [5]. Treatment modalities for VUR including conservative antimicrobial prophylaxis, ureteral reimplantation, and endoscopic injection of bulking agents into the refluxing ureter [8]. Low-grade reflux (grades 1–2) may cure naturally, whereas high-grade reflux (grades 3–5) generally necessitates surgical intervention [6].

The endoscopic injection of bulking agents, including Deflux, has emerged as an effective alternative to prolonged antibiotic therapy and ureteral reimplantation [7, 9]. This operation has become popular because to its minimally invasive characteristics and success rates of 77%–83%, accompanied by low complication rates [10]. Furthermore, it can be conducted as an outpatient operation, minimizing the necessity for extended hospital admissions. Nonetheless, problems such as ureteral blockage, albeit infrequent, have been documented [11]. The initial recorded instance of symptomatic acute ureteral blockage following subureteric Teflon injection originates from the 1990s [12]. Snodgrass documented a delayed ureteric blockage that manifested 16 weeks subsequent to Deflux injection [13]. Ureteral blockage has been noted subsequent to the administration of various bulking agents, including polyacrylate polyalcohol (Vantris®), collagen, calcium hydroxyapatite (Coaptite®), and polydimethylsiloxane (Macroplastique®) [14, 15]. The documented incidence rates of postoperative ureteral blockage differ by agent, with Deflux (dextranomer/hyaluronic acid) exhibiting an incidence of 0.5%–6.1%, polyacrylate polyalcohol (PP) 1.1%–1.6%, and polydimethylsiloxane (PDMS) 2.5%–10% [16, 17]. The etiology of ureteral obstruction subsequent to bulking agent injections is multifaceted, encompassing the type of bulking agent, the volume administered, and the technique employed. Research indicates that frequent injections elevate the likelihood of ureteral blockage [18]. The onset of obstruction might vary considerably, with instances arising as soon as a few hours post-operatively and extending up to 63 months later [19–21]. The injected area had a pronounced inflammatory response, despite the minimal volume of Deflux administered. The probable cause was acute blockage at the uretero-vesical junction. It underscores the necessity of vigilant post-procedural surveillance to swiftly detect and address problems such ureteral blockage. In summary, sub-ureteric injection of bulking agents is generally safe; nevertheless, a minority of patients may experience notable early and late problems. Acute post-injection reactions are uncommon; however, all patients must be meticulously observed and promptly assessed, particularly in the presence of stomach pain or anuria.
